# Antigen-Specific IFN-*γ* Responses Correlate with the Activity of* M. tuberculosis* Infection but Are Not Associated with the Severity of Tuberculosis Disease

**DOI:** 10.1155/2016/7249369

**Published:** 2016-11-30

**Authors:** Irina Yu. Nikitina, Alexander V. Panteleev, Ekaterina V. Sosunova, Natalya L. Karpina, Tatef R. Bagdasarian, Irina A. Burmistrova, Sofia N. Andreevskaya, Larisa N. Chernousova, Irina A. Vasilyeva, Irina V. Lyadova

**Affiliations:** ^1^Department of Immunology, Central Tuberculosis Research Institute, Yauza Alley 2, Moscow 107564, Russia; ^2^Phthisiology Department, Central Tuberculosis Research Institute, Yauza Alley 2, Moscow 107564, Russia; ^3^Department of Microbiology, Central Tuberculosis Research Institute, Yauza Alley 2, Moscow 107564, Russia

## Abstract

IFN-*γ* is a key cytokine in antituberculosis (TB) defense. However, how the levels of its secretion affect* M. tuberculosis* (*Mtb*) infection is not clear. We have analyzed associations between IFN-*γ* responses measured in QuantiFERON®-TB Gold In-tube (QFT) assay, TB disease severity, and* Mtb* infection activity. TB severity was evaluated based on the results of radiological, microbiological, and clinical examinations. Antigen-driven IFN-*γ* secretion did not correlate with TB severity. Mitogen-induced IFN-*γ* secretion correlated inversely with the form of pulmonary pathology and the area of affected pulmonary tissue; the levels of spontaneous IFN-*γ* secretion correlated with patients' age (*r* = 0.395, *p* = 0.001).* Mtb* infection activity was evaluated based on radiological data of lung tissue infiltration, destruction, dissemination or calcification, and condensation. The rate of positive QFT results and the levels of antigen-driven IFN-*γ* secretion increased in a row: patients with residual TB lesions < patients with low TB activity < patients with high TB activity. Thus, antigen-driven IFN-*γ* secretion and QFT results did not associate with TB severity but associated with the infection activity. The results suggest that quantitative parameters of IFN-*γ* secretion play a minor role in determining the course of TB disease but mirror the activity of the infectious process.

## 1. Introduction

IFN-*γ* is critical for tuberculosis (TB) protection. It is assumed that it mediates protection by stimulating macrophages for mycobacteria killing [1–3]. The concept is supported by the increased susceptibility of IFN-*γ*-deficient mice to experimental TB [4–6] and severe mycobacterial infections in humans bearing mutations in IFN-*γ*/IFN-*γ* axis [7–9]. However, several recent studies contradict this concept and suggest new roles for IFN-*γ* during TB (reviewed in [10]). In particular, in recent experimental studies IFN-*γ* was suggested to contribute to protection by inhibiting neutrophilic inflammation, whereas its role in the inhibition of* Mtb* replication was questioned [11–13]. Further, IFN-*γ* levels and the frequencies of* Mtb*-specific IFN-*γ* producing cells induced by vaccination poorly correlate with the protection against TB [14–18]. In PD-1 knockout mice, uncontrolled CD4^+^ T cell response accompanied by increased IFN-*γ* production was deleterious [19, 20]. Thus, while a complete lack of IFN-*γ* increases mice susceptibility to* Mtb* infection, it remains unclear how quantitative characteristics of IFN-*γ* responses are associated with the infection outcome.

One of the most widely used approaches to address the role of a factor in TB protection in human is to compare its expression in individuals with latent TB infection (LTBI) and patients with microbiologically confirmed sputum smear-positive TB. In this approach, individuals with LTBI are considered as developing effective immune responses, while TB patients as being unable to efficiently contain* Mtb* infection. Comparisons of IFN-*γ* responses during LTBI and TB have resulted in contradictory results. Some authors showed increased plasma levels of IFN-*γ* or increased frequencies of circulating IFN-*γ* producing cells in TB patients compared to LTBI [21]. Other groups reported reduced capacity of peripheral blood mononuclear cells from TB patients for antigen-driven secretion of IFN-*γ* [22–24] and suggested that IFN-*γ* deficiency contributes to TB pathogenesis.

These inconsistencies can be explained by the differences between the studies in methodology (i.e., antigens used to stimulate cells and stimulation procedures) and patient spectrum. Another possible explanation takes into account the complexity of the relationships between IFN-*γ* responses and* Mtb* infection activity. Indeed, the extent of IFN-*γ* responses controlled by genetic and/or other* Mtb* infection independent factors (e.g., nutritional and stressful) affects the outcome of* Mtb* infection. In this model, the lower the IFN-*γ* response is, the higher the infection activity would be. On the other hand, active disease and* Mtb*-derived signals stimulate T cell immunity and IFN-*γ* secretion. Thus, the more active the infection is, the higher the immune response should be. Next, chronic infection and persistent antigenic stimulation induce T cell exhaustion dampening IFN-*γ* secretion [25]. Additional complexity comes from the fact that TB disease is highly heterogeneous by its manifestations and severity. This heterogeneity is rarely taken into account, resulting in a poor understanding of how IFN-*γ* associates with TB severity and outcomes.

In this study we have used a standardized procedure of QuantiFERON-TB Gold In-Tube (QFT) assay to analyze the extent of IFN-*γ* responses in TB patients and examine how quantitative characteristics of these responses are associated with the activity and the severity of* Mtb* infection in human.

## 2. Materials and Methods

### 2.1. Ethics

All studies were conducted in accordance with the principles expressed in the Helsinki Declaration, approved by the IRB #1 of the Central Tuberculosis Research Institute and performed between years 2010 and 2015.

### 2.2. Study Participants

**Figure 1:**
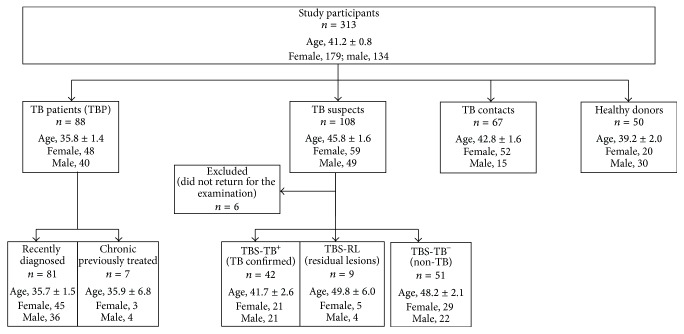
Study population.

A total of 313 participants were enrolled in the study. They formed the following groups: TB patients (TBP), TB suspects (TBS),* Mtb*-exposed healthy individuals having high risk of LTBI (TB contacts, TBC), and healthy donors with no known records of* Mtb* exposure (HD) (Figure 1). All participants gave written informed consent to participate in the study.

Patients from TBP group (*n* = 88; age 35.8 ± 1.4; 48 women, 40 men) underwent treatment in the Central Tuberculosis Research Institute, Moscow (CTRI). Eighty-two patients were diagnosed for TB based on clinical and radiographic evidences of TB and identification of* Mtb* and/or* Mtb* DNA in the sputum. In six patients, the diagnosis was based on clinical and radiographic evidences of TB and positive response to anti-TB therapy (i.e., positive clinical and radiographic dynamics assessed by independent clinicians 2 months following the treatment). In these patients, final diagnosis was made after the performance of QFT; all clinicians were blind to QFT results. Among 88 TB patients, 81 patient had recently diagnosed TB; 7 patients had chronic TB (>1 year) and had received several courses of therapy by the time of analysis. In TBP with recently diagnosed TB, QFT was performed within the first two weeks of antituberculosis therapy.

TBS (*n* = 108; age 45.8 ± 1.6 years; 59 women, 49 men) were examined at the CTRI for the diagnostic purposes without hospitalization. In this group, QFT was performed at diagnosis. Final diagnosis was made by clinicians not aware of the results of QFT. The diagnosis was based on the results of microbiological, radiological, and clinical examinations. Of 108 TBS, 42 patients were diagnosed for active pulmonary TB (TBS-TB^+^), 9 patients had residual TB lesions (TBS-RL); 51 patient had non-TB pulmonary diseases (TBS-TB^−^, i.e., sarcoidosis, pneumonia, bronchitis, cancer, and chronic obstructive pulmonary disease). Six patients did not return for the examination, had undetermined final diagnosis, and were excluded from the analysis.

The group of TBC (*n* = 67; age 42.8 ± 1.6 years; 52 women, 15 men) included CTRI employees who had been working in tight contacts with TBP for at least 1 year (9.1 ± 1.1 years) and had no clinical or radiographic evidences of TB.

The group of HD (*n* = 50; age 39.2 ± 2.0 years; 20 women, 30 men) included participants with no records of* Mtb* exposure.

All TBP were HIV-seronegative. In TBS, TBC, and HD HIV status was not known as testing was not routinely offered to these study populations.

### 2.3. QuantiFERON-TB Gold In-Tube

QFT was performed according to the manufacturer protocol (Cellestis Ltd., Carnegie, Australia; QIAGEN, Valencia, CA, USA). Briefly, venous blood was drawn into NIL, AG, and MIT tubes containing no antigen,* Mtb* antigens (ESAT-6; CFP-10; TB 7.7), and mitogen, respectively. Tubes were hand-shaken and incubated at 37°C for 24 h. Samples were centrifuged and plasma was collected and stored at −20°C until analysis (7–14 days). At the day of analysis, samples were thawed, and ELISA was conducted according to the manufacturer's instructions. Optical densities were measured using Sunrise™ reader and Magellan™ software (Tecan Group Ltd.., Switzerland). Concentration of IFN-*γ* in each tube and the results of the assay were determined using “QuantiFERON-TB Gold Analysis Software” (Cellestis Ltd.) and interpreted as specified by the manufacturer. Due to a limited linear range of ELISA reader, values greater than 10 IU/mL were assigned a value of 10 IU/mL, the upper limit of the standard curve, as previously suggested by other authors [26, 27]. Because the truncation could impact the results of statistical analysis, samples from patients having high levels of IFN-*γ* in AG tubes (>10 IU/mL) were diluted, QFT was repeated for all probes (i.e., NIL, AG, and MIT), and results were reanalyzed (shown in Supplementary Figures in Supplementary Material available online at http://dx.doi.org/10.1155/2016/7249369).

### 2.4. Statistical Analysis

Statistical analysis was performed using Prizm 4.0 (GraphPad Software Inc.) and R-studio. In the text, all numeric data are shown as means with standard error (SE). Differences between independent variables were analyzed using nonparametric Mann-Whitney test for two variables and one-way ANOVA on ranks (Kruskal-Wallis test with Dunn's posttest) (GraphPad Software). Differences with *p* value < 0.05 were considered significant. Correlations between variables were analyzed using Spearman test with Benjamini-Hochberg correction (R-studio).

## 3. Results

### 3.1. Clinical, Microbiological, and Radiological Characteristics of TB Patients

Pulmonary TB disease has multifarious manifestations that mirror diverse aspects of disease pathology and severity. To examine associations between IFN-*γ* and TB severity, we evaluated the following characteristics of TB disease: the form of pulmonary pathology, the degree of pulmonary destruction, disease extent (pulmonary area affected by TB process), bacteria excretion, and intoxication. All TB characteristics were evaluated and scored by independent radiologists, microbiologists, and clinicians who were unaware of the results of QFT. To ensure the accuracy of the evaluations, only patients undergoing treatment in the CTRI and being under prolonged observation were included in the analysis. The characteristics of TB disease were scored as follows.

The forms of pulmonary pathology: score 1: tuberculoma; score 2: TB infiltrate; score 3: cavitary TB; score 4: disseminated TB; score 5: caseous pneumonia.

TB extent was scored based on the area of the lung tissue affected by TB process, that is, score 1: the process affected one to three segments of the lung; score 2: the process affected four or more segments in different lobes of the lung or one-two whole lobe(s); score 3: three lobes in different lungs; score 4: one whole lung or both lungs.

The degree of lung tissue destruction was scored based on the number and size of destructive (lucent) foci, that is, score 1: no foci; score 2: one small (<2 cm diameter) focus; score 3: several small foci or one large (≥2 cm) transparent focus; score 4: multiple foci of which at least one was large.

The presence of* Mtb* in the sputum and the level of bacteria excretion were evaluated based on the results of sputum smear (auramine-rhodamine smear microscopy), sputum culture (BACTEC radiometric method, Becton Dickinson, Sparks, MD, USA, or solid media culture), and real-time IS6110-based PCR (Amplitub-Rv, Syntol, Moscow, Russia) assays. The results were scored as follows: score 1: no acid-fast bacilli (AFB) in sputum smear plus negative sputum culture/BACTEC and PCR results; score 2: 0–9 AFB per 100 view fields and positive result of sputum culture/BACTEC or PCR; score 3: 10–99 AFB per 100 view fields and positive sputum culture/BACTEC results; score 4: 1–10 AFB in one view field and positive sputum culture/BACTEC results; score 5: more than 10 AFB per one view field and positive sputum culture/BACTEC results.

The degree of intoxication was scored from 1 to 4 based on the presence of systemic intoxication symptoms (fatigue, asthenia, night sweating, caught, and fever): score 1: no symptoms; score 2: one to two symptoms without fever; score 3: several symptoms plus subfebrile body temperature; score 4: several symptoms and febrile temperature.

The patients displayed the great variability regarding the above manifestations of TB disease and their severity (Table 1).

### 3.2. The Levels of* Mtb*-Driven IFN-*γ* Responses Are Not Associated with TB Severity

To assess IFN-*γ* responses, we examined TBP for the results of QFT (i.e., positive or negative) and the levels of IFN-*γ* secretion in unstimulated (NIL),* Mtb*-antigen stimulated (AG), and mitogen-stimulated (MIT) probes. Positive QFT results were registered in 63 out of 88 TBP (71.6%). We next correlated the results with the characteristics of TB disease.

Spearman correlation analysis with Benjamini-Hochberg correction showed that QFT results and the levels of antigen-driven IFN-*γ* secretion (i.e., AG and AG-NIL) did not correlate with any characteristic of TB severity (Table 2). Significant correlations were found only between (i) the levels of spontaneous IFN-*γ* production (NIL probes) and patient' age (*r* = 0.391, *p* = 0.001) and (ii) the levels of mitogen-induced IFN-*γ* production (MIT probes) and the form of pulmonary pathology (*r* = −0.352, *p* = 0.004) and TB extent (*r* = −0.395, *p* = 0.001).

The results indicated that the basal production of IFN-*γ* increases with age and that in patients having more extended pulmonary TB IFN-*γ* responses are suppressed in antigen-independent manner. On the other hand, the levels of IFN-*γ* secretion driven by* Mtb*-specific antigens appeared to be not associated with TB severity.

**Figure 2:**
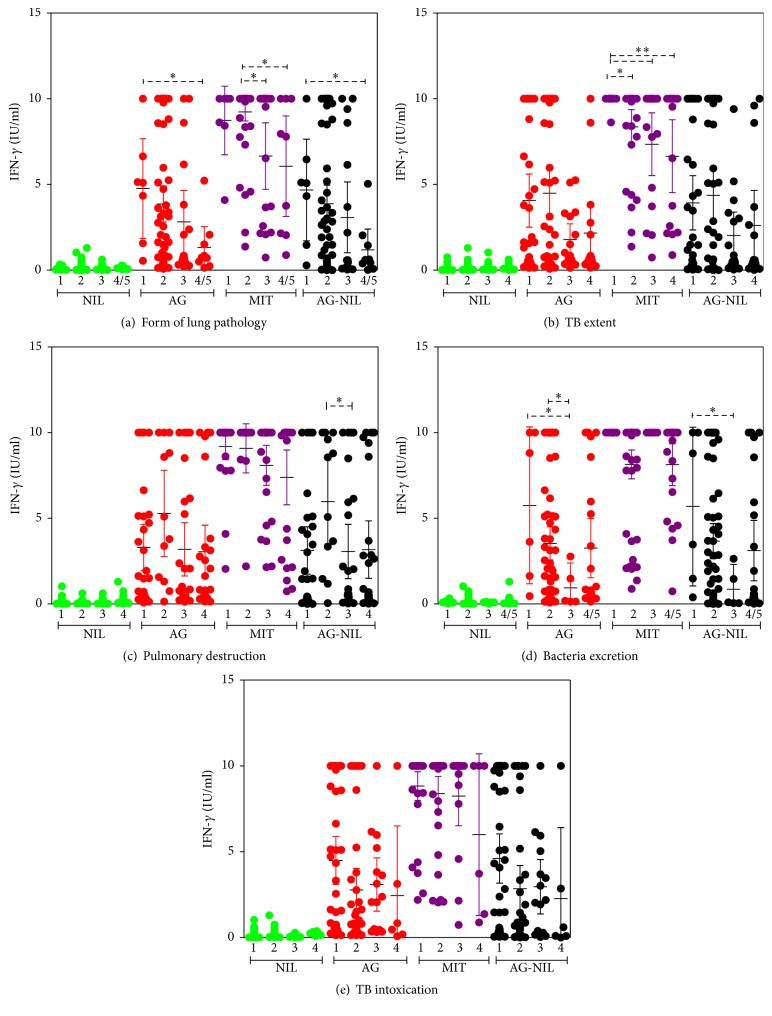
The levels of IFN-*γ* secretion measured in QFT assay in patients having different severity of TB disease. Patients were grouped based on the forms of pulmonary pathology (a), TB extent (b), the degree of pulmonary destruction (c), bacteria excretion (d), and intoxication (e). Each TB characteristic was scored as described in Section 3.1. IFN-*γ* concentrations greater than 10 IU/mL were assigned a value of 10 IU/mL. Figures on *X*-axis indicate scores. Shown are the differences determined in Mann-Whitney test (^*∗*^ *p* < 0.05; ^*∗∗*^ *p* < 0.01). In Kruskal-Wallis analysis with Dunn's posttest, the differences between the groups were insignificant.

Because associations between IFN-*γ* and TB severity are complex and may be nonlinear, we next divided all patients into groups based on the scores for each TB characteristic and analyzed IFN-*γ* responses in each group. TBP having similar severity scores were highly heterogeneous with regard to IFN-*γ* responses (Figure 2). The differences between the groups in the levels of antigen-driven IFN-*γ* secretion were not very strong. In Mann-Whitney test, significant differences with *p* < 0.05 were detected between patients with disseminated TB/caseous pneumonia and patients with tuberculoma (scores 4/5 and 1, resp.), patients with high pulmonary destruction and patients with mild destruction (scores 3 and 2, resp.), and patients with mild bacteria excretion and patients with low bacteria excretion or the absence of bacteria in the sputum (scores 3, 2, and 1, resp., Figures 2(a), 2(c), and 2(d)). However, in Kruskal-Wallis test with Dunn's posttest, all comparisons were insignificant. Of note, in some patients, the levels of IFN-*γ* secretion in AG tubes exceeded the upper limit of QFT test and were assigned a value of 10 IU/mL. Because the truncation could affect the results of statistical analysis, and antigen-driven IFN-*γ* secretion was in the main focus of our study, we next diluted these samples and repeated QFT test. The differences between the groups were similar to those described above (Supplementary Figure  1). It should also be noted that some associations between antigen-driven IFN-*γ* responses and TB characteristics tended to be second-order polynomial. For example, the levels of IFN-*γ* were higher in patients having the least and the highest degrees of bacteria excretion compared to patients with mild bacteria excretion (*Y* = 10.64 + *X* ^4.95^ + *X* ^0.76^). This supports the concept that IFN-*γ* response both influences and is influenced by TB disease characteristics.

### 3.3. The Rate of Positive QFT Results Mirrors* Mtb* Infection Activity

**Figure 3:**
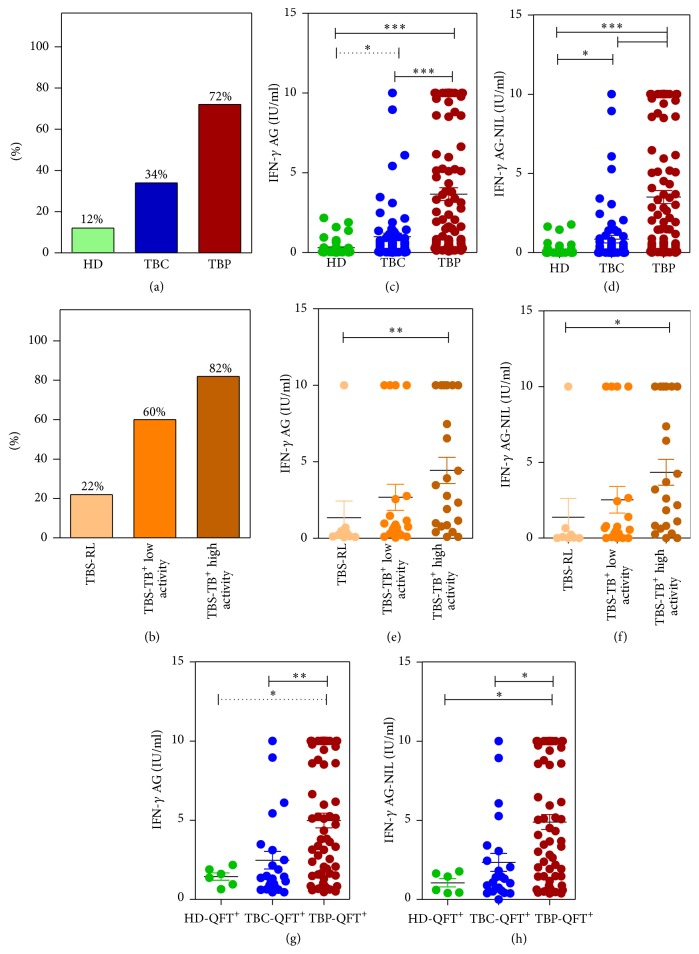
The performance of QFT in HD, TBC, and patients with different TB activity. (a) Percent of positive QFT results in groups of study participants having different risk of* Mtb* infection. (b) Percent of positive QFT results in TBS having different activity of TB disease. (c–f) The levels of antigen-driven IFN-*γ* secretion in groups of participants with different risk (c, d) and activity (e, f) of TB infection. (g, h) The levels of antigen-driven IFN-*γ* secretion in HD, TBC, and TBP with positive QFT results. IFN-*γ* concentrations greater than 10 IU/mL were assigned a value of 10 IU/mL. HD, healthy donors; TBC, TB contacts; TBP, TB patients; TBS-RL, TB suspects having residual TB lesions in the lungs; TBS-TB^+^-low activity, TB suspects diagnosed for pulmonary TB and having radiological signs of low TB activity; TBS-TB^+^ high activity, TB suspects diagnosed for pulmonary TB and having radiological signs of high TB activity. Shown are the differences determined in Mann-Whitney test (^*∗*^ *p* < 0.05; ^*∗∗*^ *p* < 0.01; ^*∗∗∗*^ *p* < 0.001). Solid lines indicate differences that were also significant in Kruskal-Wallis test with Dunn's posttest. Dotted lines, differences significant in Mann-Whitney and insignificant in Kruskal-Wallis test with Dunn's posttest.

We next evaluated IFN-*γ* production in TBC (i.e., participants having high risk of LTBI) and HD (i.e., participants having low risk of TB infection). Positive QFT results were registered in 23 out of 67 TBC (34%) and in 6 out of 50 HD (12%). Thus, the rate of positive QFT results increased in a row: HD < TBC < TBP, that is, on a group level, mirrored the probability/risk of TB infection (Figure 3(a)). The levels of antigen-driven IFN-*γ* secretion also were higher in TBP compared to TBC and HD (Figures 3(c) and 3(d) and Supplementary Figures  2C and 2D).

Radiologically, all TBP included in the study had signs of high disease activity, that is, signs of tissue infiltration, destruction, and/or dissemination. Aiming to analyze whether IFN-*γ* responses are associated with the activity of* Mtb* infection, we next evaluated IFN-*γ* production in TBS who supposedly could have different activity of TB disease.

TBS were examined at the CTRI to clarify the diagnosis. Totally, 108 TBS were enrolled in the study. Subsequently, they were diagnosed for active TB (TBS-TB^+^), residual TB lesions (TBS-RL), or non-TB pulmonary diseases (TBS-TB^−^) (Figure 1). Radiological examination of TBS-TB^+^ showed that they differed by the activity of TB process: 22 patients had high TB activity (i.e., they had radiological sings of tissue infiltration, destruction, and/or dissemination), whereas 20 patients exhibited evidences of low TB activity (i.e., they had radiological sings of calcification and condensation of pulmonary lesions). Residual TB lesions were characterized by a complete calcification and/or scarring of pulmonary lesions.

Positive QFT results were registered in 18 out of 22 patients with high TB activity (82%), 12 out of 20 patients with low TB activity (60%), 2 out of 9 TBS-RL (22%, one patient had undetermined QFT results), and 7 out of 51 TBS-TB^−^ (14%, Figure 3(b)). Thus, the rate of positive QFT results increased in a row: TBS-RL < TBS-TB^+^ with low TB activity < TBS-TB^+^ with high TB activity (Figure 3(b), Supplementary Figure  2B). The levels of antigen-driven IFN-*γ* secretion also were higher in patients with more active TB disease (Figures 3(e) and 3(f); Supplementary Figures  2E and 2F).

We then scored* Mtb* infection activity (score 1: TBS-RL, score 2: TBS-TB^+^ with low TB activity; score 3: TBS-TB^+^ with high TB activity) and analyzed its correlation with the levels of antigen-driven IFN-*γ* secretion (AG-NIL). The correlation was significant (*r* = 0.419, *p* = 0.003 for IFN-*γ* levels truncated at 10 IU/mL, *r* = 0.440, *p* = 0.001 for real IFN-*γ* concentrations; see Section 2).

An association between the levels of antigen-driven IFN-*γ* secretion and infection activity was indirectly confirmed when we compared IFN-*γ* responses in HD, TBC, and TBP with positive QFT results (i.e., in HD-QFT^+^, TBC-QFT^+^, and TBP-QFT^+^). In this comparison, HD-QFT^+^ and TBC-QFT^+^ were considered as individuals with LTBI. Patients with active TB (TBP-QFT^+^) had higher levels of antigen-driven IFN-*γ* secretion compared to participants with LTBI (Figures 3(g) and 3(h)).

Overall, participants with more active* Mtb* infection developed higher antigen-driven IFN-*γ* response compared to participants with less active infection.

## 4. Discussion

IFN-*γ* is a key cytokine in anti-TB defense. Its complete lack leads to extremely severe mycobacterial infections [4, 5, 7–9]. In the absence of mutations in genes encoding IFN-*γ*/IFN-*γ* axis proteins, IFN-*γ* is readily produced in response to host exposure to* Mtb*. However, whether the levels of IFN-*γ* secretion affect* Mtb* infection activity and outcomes is not fully clear.

Besides the fact that IFN-*γ* contributes to protection, it is used for screening purposes and the identification of* Mtb* infection in IGRA assays. Thus, understanding the relationships between the levels of IFN-*γ* secretion and the status of* Mtb* infection is important for both diagnostic purposes and the development of host-directed therapy.

In this study we have used QFT assay to quantify the levels of basal, antigen-driven, and mitogen-induced IFN-*γ* secretion by blood cells isolated from TBP, TBC, HD, and TBS diagnosed for TB or non-TB pulmonary diseases. The rate of positive QFT results and the levels of antigen-driven IFN-*γ* secretion increased in a row: HD (12%) < TBC (34%) < TBP (72%), that is, mirrored the probability/risk of* Mtb* infection. QFT assay is based on the detection of memory T cells that are generated and maintained in response to TB exposure and* Mtb* infection. It is assumed that QFT identifies* Mtb* infection and associates with the level of* Mtb* exposure but fails to distinguish active TB and LTBI [28, 29]. Our data on the rate of positive QFT results in HD, TBC, and TBP groups are in a good line with this concept.

The performance of QFT during active TB has been addressed in multiple studies [28–32]. Most of the studies reported suboptimal sensitivity of the test for active TB. Our results are in line with these observations. It is usually assumed that suboptimal sensitivity of QFT for active TB is due to the immune suppression developed as a result of severe TB disease. Our data do not support this concept. In the study, we paid a special attention to the careful examination of TB patients and thorough assessment of TB disease characteristics, including microbiological (i.e., the degree of bacteria excretion), radiological (i.e., the form of pulmonary pathology, TB extent, and the degree of pulmonary destruction), and clinical (i.e., the degree of intoxication). We found no strong correlation between the levels of antigen-driven IFN-*γ* secretion and TB severity. When TBP were divided into groups based on TB severity scores, significant differences in IFN-*γ* secretion were observed mainly between patients having most severe and least severe TB (e.g., between patients with tuberculoma and disseminated TB/caseous pneumonia, but not between patients having tuberculoma, TB infiltrate, and cavitary TB). Moreover, even between the polar groups, the differences were not highly significant (i.e., *p* < 0.05 in Mann-Whitney test and insignificant in Kruskal-Wallis test with Dunn's posttest). Thus, TB severity was not tightly associated with the levels of antigen-specific IFN-*γ* response. This suggests that suboptimal QFT sensitivity for active TB can hardly be attributed to the immune suppression developed in severely-ill patients and that quantitative parameters of systemic IFN-*γ* responses play minor role in determining TB severity (unless there is a complete lack of IFN-*γ*/IFN-*γ* axis proteins, which leads to severe consequences for the host [7–9]).

In contrast to antigen-driven IFN-*γ* secretion, mitogen-induced secretion correlated significantly and inversely with TB severity, in particular, with TB extent. These results indicate that severe TB disease induces immune suppression/anergy but in antigen-independent manner.

While QFT performance did not associate with TB severity, it appeared to be associated with the activity of TB disease. In our study, the rates of positive QFT results and the levels of antigen-driven IFN-*γ* secretion increased in a row: TBS-RL < TBS-TB^+^ with low TB activity < TBS-TB^+^ with high TB activity: that is, they were higher in patients with more active TB disease. These results extend existing observations on QFT performance and raise a question on the sensitivity of QFT for LTBI (i.e., “inactive”* Mtb* infection). In our study, only 34% of individuals working in tight contact with TB patients had positive QFT results. Whether QFT-negative TBC were free from LTBI is an open question. One possibility is that QFT-negative TBC were uninfected. Another possibility is that QFT does not identify LTBI with 100% sensitivity. Our data on suboptimal QFT sensitivity for active TB along with a better performance of QFT in TBS with more active disease indirectly support the second hypothesis. In the absence of a gold standard for LTBI, it is difficult to conclude which of the explanations is more likely. However, the possibility that at least some individuals with negative IGRA may harbor LTBI should be taken into account.

## 5. Conclusions

To summarize, in this study we have demonstrated that (i) in TB patients the levels of antigen-driven IFN-*γ* secretion and QFT results are not associated with TB severity evaluated in microbiological and radiological tests or clinical examinations; (ii) the levels of antigen-driven IFN-*γ* secretion are associated with the activity of TB disease. The results suggest that quantitative parameters of IFN-*γ* responses mirror the activity of* Mtb* infection but play a minor role in determining the course and severity of TB disease. The reasons for the suboptimal QFT sensitivity for active TB disease are yet to be determined.

## Supplementary Material

Supplementary Figure 1: The levels of IFN-γ secretion measured in QFT assay in patients having different severity of TB disease. Patients were grouped as based on the characteristics of TB disease, as described in Figure 2. Each TB characteristic was scored as described in the section 3.1. The levels of IFN-γ were determined using QFT assay. The values of IFN-γ in MIT samples greater than 10 IU/mL were assigned a value of 10 IU/mL, the upper limit of the standard curve in QFT. The exact levels of IFN-γ secretion in AG tubes were determined (samples from patients having high levels of IFN-γ in AG tubes were diluted and QFT was repeated for all probes, i.e., NIL, AG, and MIT).
Figures on X-axis indicate scores. In ANOVA, the differences between the groups were insignificant. Shown are the differences determined in Mann-Whitney test (∗, p<0.05; ∗∗, p<0.01; ∗∗∗, p<0.001).
Supplementary Figure 2: The performance of QFT in HD, TBC, and patients with different TB activity. The levels of IFN-γ were determined using QFT assay. The values of IFN-γ in MIT samples greater than 10 IU/mL were assigned a value of 10 IU/mL, the upper limit of the standard curve in QFT. The exact levels of IFN-γ secretion in AG tubes were determined (samples from patients having high levels of IFN-γ in AG tubes were diluted and QFT was repeated for all probes, i.e., NIL, AG, and MIT). For other details, see footnote to Figure 3.

## Figures and Tables

**Table 1:** Heterogeneity of TB patients included in the study.

TB characteristic	Number of TBP having the indicated scores				
	Score 1	Score 2	Score 3	Score 4	Score 5
Forms of pulmonary pathology	7	55	17	7	2
TB extent	27	30	16	15	NA
The degree of pulmonary destruction	27	12	25	24	NA
The degree of bacteria excretion	6	55	5	19	3
The degree of intoxication	34	33	15	6	NA

NA: not applicable.

**Table 2:** Correlations between IFN-*γ* responses and TB disease severity.

TB characteristics	rho/*p*-value	IFN-*γ*_NIL_	IFN-*γ*_AG_	IFN-*γ*_MIT_	IFN-*γ*_AG-NIL_	QFT results
Forms of pulmonary pathology	rho	0.045	−0.239	−**0.352 **	−0.235	−0.091
	*p* value	0.713	0.080	**0.004**	0.084	0.521

TB extent	rho	0.050	−0.165	−**0.395**	−0.156	−0.060
	*p* value	0.694	0.253	**0.001**	0.286	0.650

The degree of pulmonary destruction	rho	0.108	−0.080	−0.226	−0.063	−0.024
	*p* value	0.485	0.576	0.095	0.643	0.842

The degree of bacteria excretion	rho	0.077	−0.201	−0.125	−0.209	−0.213
	*p* value	0.579	0.144	0.398	0.125	0.121

The degree of intoxication	rho	0.084	−0.178	−0.195	−0.173	−0.099
	*p* value	0.558	0.212	0.156	0.227	0.497

Age	rho	**0.391**	−0.020	0.101	−0.066	−0.094
	*p* value	**0.001**	0.855	0.497	0.633	0.516

Correlations were analysed using nonparametric Spearman test with Benjamini-Hochberg correction. Significant correlations are shown in bold.
