# Supplementary figures and images for: Antigen-Specific IFN-γ Responses Correlate with the Activity of M. tuberculosis Infection but Are Not Associated with the Severity of Tuberculosis Disease
Source: J Immunol Res. 2016 Nov 30;2016:7249369. doi: 10.1155/2016/7249369 (PMC5155109; doi:10.1155/2016/7249369)

Supplement Figure 1

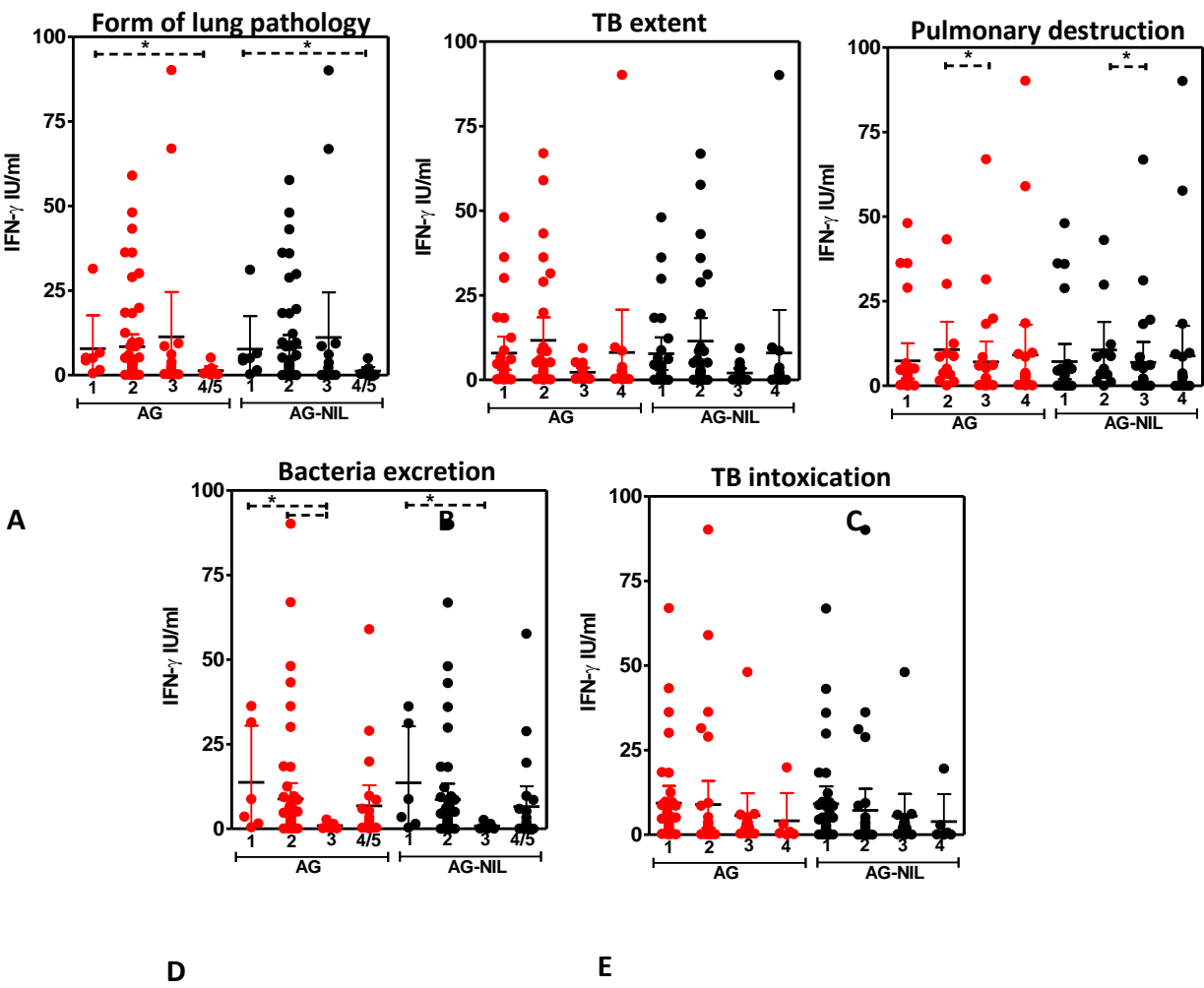

Supplement Figure 2

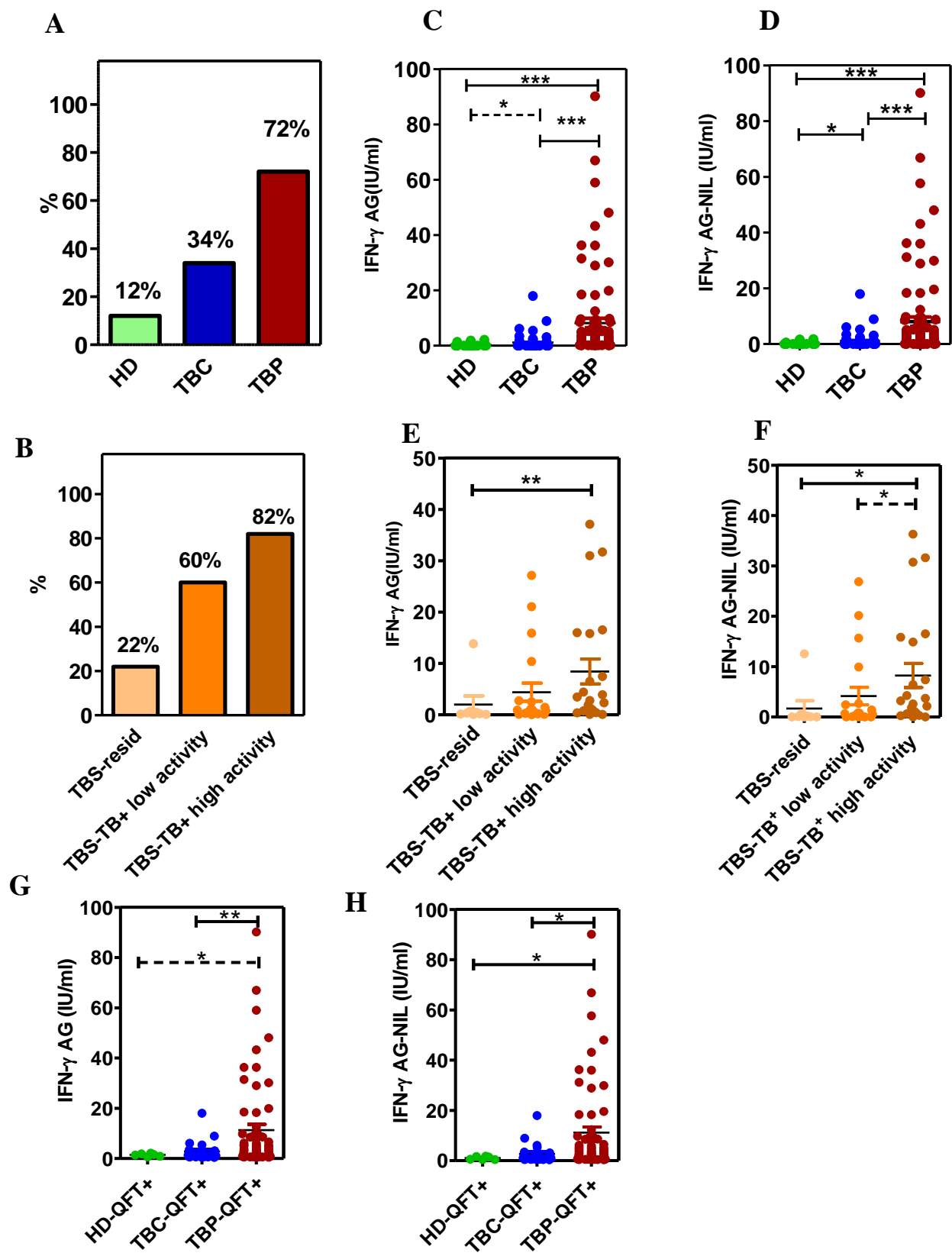

Supplement: Supplementary file 1 — Supplementary Figure 1: The levels of IFN-γ secretion measured in QFT assay in patients having different severity of TB disease. Patients were grouped as based on the characteristics of TB disease, as described in Figure 2. Each TB characteristic was scored as described in the section 3.1. The levels of IFN-γ were determined using QFT assay. The values of IFN-γ in MIT samples greater than 10 IU/mL were assigned a value of 10 IU/mL, the upper limit of the standard curve in QFT. The exact levels of IFN-γ secretion in AG tubes were determined (samples from patients having high levels of IFN-γ in AG tubes were diluted and QFT was repeated for all probes, i.e., NIL, AG, and MIT). Figures on X-axis indicate scores. In ANOVA, the differences between the groups were insignificant. Shown are the differences determined in Mann-Whitney test (∗, p<0.05; ∗∗, p<0.01; ∗∗∗, p<0.001). Supplementary Figure 2: The performance of QFT in HD, TBC, and patients with different TB activity. The levels of IFN-γ were determined using QFT assay. The values of IFN-γ in MIT samples greater than 10 IU/mL were assigned a value of 10 IU/mL, the upper limit of the standard curve in QFT. The exact levels of IFN-γ secretion in AG tubes were determined (samples from patients having high levels of IFN-γ in AG tubes were diluted and QFT was repeated for all probes, i.e., NIL, AG, and MIT). For other details, see footnote to Figure 3. [file 7249369.f1.pdf]
